# Autonomous navigation of a magnetic colonoscope using force sensing and a heuristic search algorithm

**DOI:** 10.1038/s41598-021-95760-7

**Published:** 2021-08-13

**Authors:** Hao-En Huang, Sheng-Yang Yen, Chia-Feng Chu, Fat-Moon Suk, Gi-Shih Lien, Chih-Wen Liu

**Affiliations:** 1 Department of Electrical Engineering, National Taiwan University, No. 1, Sec. 4, Roosevelt Rd., Taipei, 10617 Taiwan (R.O.C.); 2 Division of Gastroenterology, Department of Internal Medicine, Taipei Municipal Wan Fang Hospital, Taipei Medical University, Taipei, Taiwan (R.O.C.); 3 Department of Internal Medicine, School of Medicine, College of Medicine, Taipei Medical University, Taipei, Taiwan (R.O.C.)

**Keywords:** Colonoscopy, Large intestine, Biomedical engineering

## Abstract

This paper presents an autonomous navigation system for cost-effective magnetic-assisted colonoscopy, employing force-based sensors, an actuator, a proportional–integrator controller and a real-time heuristic searching method. The force sensing system uses load cells installed between the robotic arm and external permanent magnets to derive attractive force data as the basis for real-time surgical safety monitoring and tracking information to navigate the disposable magnetic colonoscope. The average tracking accuracy on magnetic field navigator (MFN) platform in x-axis and y-axis are 1.14 ± 0.59 mm and 1.61 ± 0.45 mm, respectively, presented in mean error ± standard deviation. The average detectable radius of the tracking system is 15 cm. Three simulations of path planning algorithms are presented and the learning real-time A* (LRTA*) algorithm with our proposed directional heuristic evaluation design has the best performance. It takes 75 steps to complete the traveling in unknown synthetic colon map. By integrating the force-based sensing technology and LRTA* path planning algorithm, the average time required to complete autonomous navigation of a highly realistic colonoscopy training model on the MFN platform is 15 min 38 s and the intubation rate is 83.33%. All autonomous navigation experiments are completed without intervention by the operator.

## Introduction

Colonoscopy screening programs are considered the gold standard examination procedure for colorectal cancer (CRC), providing the ability to carefully inspect the entire gastrointestinal (GI) tract and reduces colorectal cancer incidence^1,2^. However, the intrusive nature of the operation, the rigidity of the device, and the need to maneuver it manually can make the experience highly unpleasant for the recipient^3^. The development of capsule endoscopy (CE) has made the screening process far more tolerable^4,5^; however, the passive movement of the capsule endoscope through the GI tract greatly hinders the analysis of data after the examination^6^ and imposes the risk of capsule retention within a tumor or a narrow area^7,8^.

Numerous locomotion methods have been developed for CE navigation^9^. The fact that manual navigation systems are highly susceptible to human error and require that operators undergo extensive training has prompted development in autonomous locomotion systems for endoscopy^10–12^. Therefore, several articles on autonomous navigation of colonoscopy have shown the value of this field^10–14^. However, in order to obtain the precise position of the endoscope in the human body in real-time, the complex sensing structure in the endoscope and computation algorithms are inevitable^15–17^. In addition, several researches have shown that postendoscopic infections from patient-to-patient transmission are more frequent than commonly expected^18–20^. The results also suggest infection-related complications occurred within couple of days. As a consequence, the complex structure of the endoscope and the value of disposable colonoscopy have prompted us to develop another method so that cheaper endoscopes can be used for the benefit of the general public.

**Figure 1:**
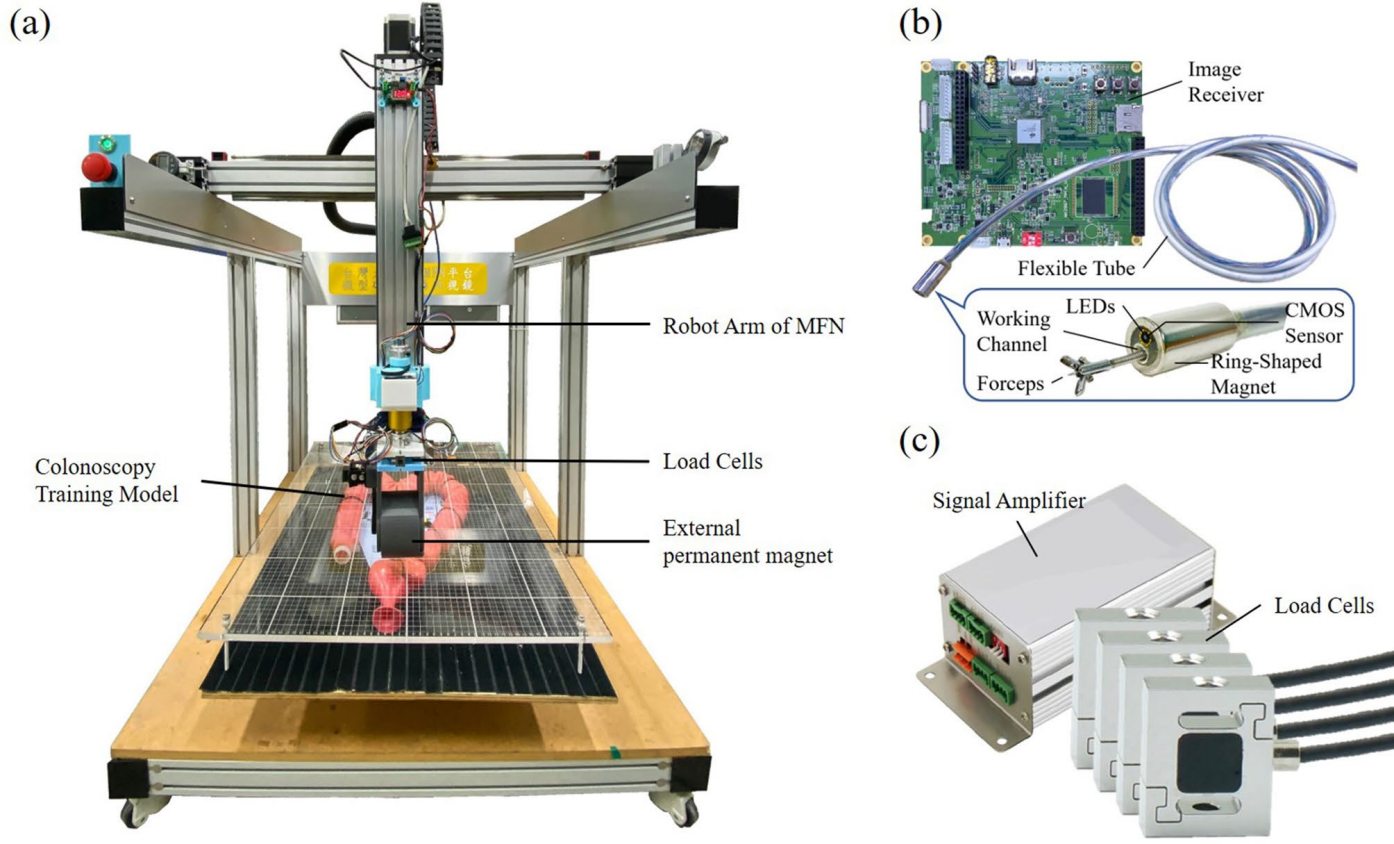
The architecture of magnetic-assisted colonoscopy (MAC) system. (**a**) Magnetic field navigator (MFN). (**b**) Disposable magnetic colonoscope (MC) and its receiver. (**c**) Tension/compression load cells and their signal amplifier.

**Figure 2:**
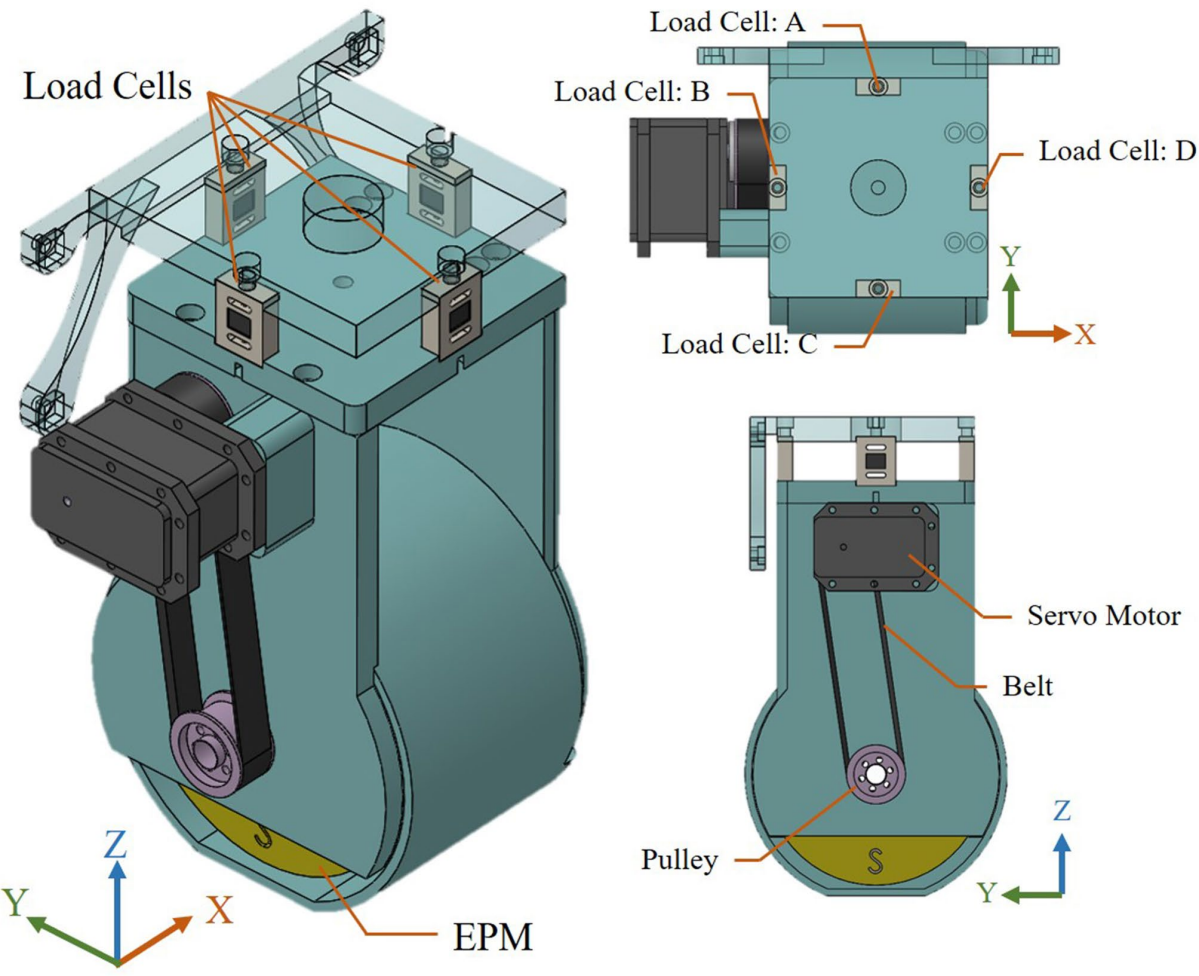
Schematic illustration showing the architecture of proposed force sensing system, featuring load cells installed between the robot arm and the container holding a large permanent magnet.

In this work, we developed a magnetic-assisted colonoscopy (MAC) system featuring force-based sensing technology and applied the learning real-time A* (LRTA*) searching scheme using a cost-effective colonoscope to facilitate autonomous navigation within a highly realistic colonoscopy training model. The work is done with cost-effective hardware and the feasibility is assured by experimental results. Force-based sensing technology is used as control input and decreased the uncertainty in applied force^21–23^. In the current study, endoscope tracking was firstly achieved using force-based sensing technology, wherein load cells were mounted in the joint of a robotic arm (see Figs. 1a and 2) to sense attractive forces associated with capsule endoscopes in three-dimensions. This scheme enables the real-time tracking of an interior permanent magnet (IPM) in magnetic colonoscope (MC) with a high degree of accuracy by balancing the four force vectors from load cell module without further add-ons to the system. In doing so, the proposed method won’t have to figure out the actual 2D position of IPM and the computation can be simplified. Additionally, the ability to monitor attractive forces also helps to prevent damage to human tissue resulting from excessive force.

## Results

### Positioning of IPM: detectable range and maximum attraction of positioning IPM

The purpose of this experiment is to validate the feasibility of the proposed tracking system based on force-based sensing technology. Table 1 lists the maximum attractive forces and effective detectable range at four different vertical distances from external permanent magnet (EPM) to MC. The theoretical maximum attractive force is related directly to the safety of the magnetic tracking system. The use of load cells makes it easier to obtain the attractive force imposed on the organ by the MC’s tip. Overall, the peak force in each of these experiments was less than that of conventional colonoscopy presented by Korman et al.^24^ and Plooy et al.^25^ which were approximately 33.5 N and 27.5 N, respectively. The detectable range also shows that it is sufficient to the tracking system.

**Table 1:** Maximum attractive force and effective detectable range in proposed tracking system.

Vertical distance from EPM to MC (cm)	Detectable radius (cm)	Maximum attraction (gF)
10	20	63
7.5	18	125
5	12	220
2.5	10	612

### Positioning of IPM: tracking accuracy

**Figure 3:**
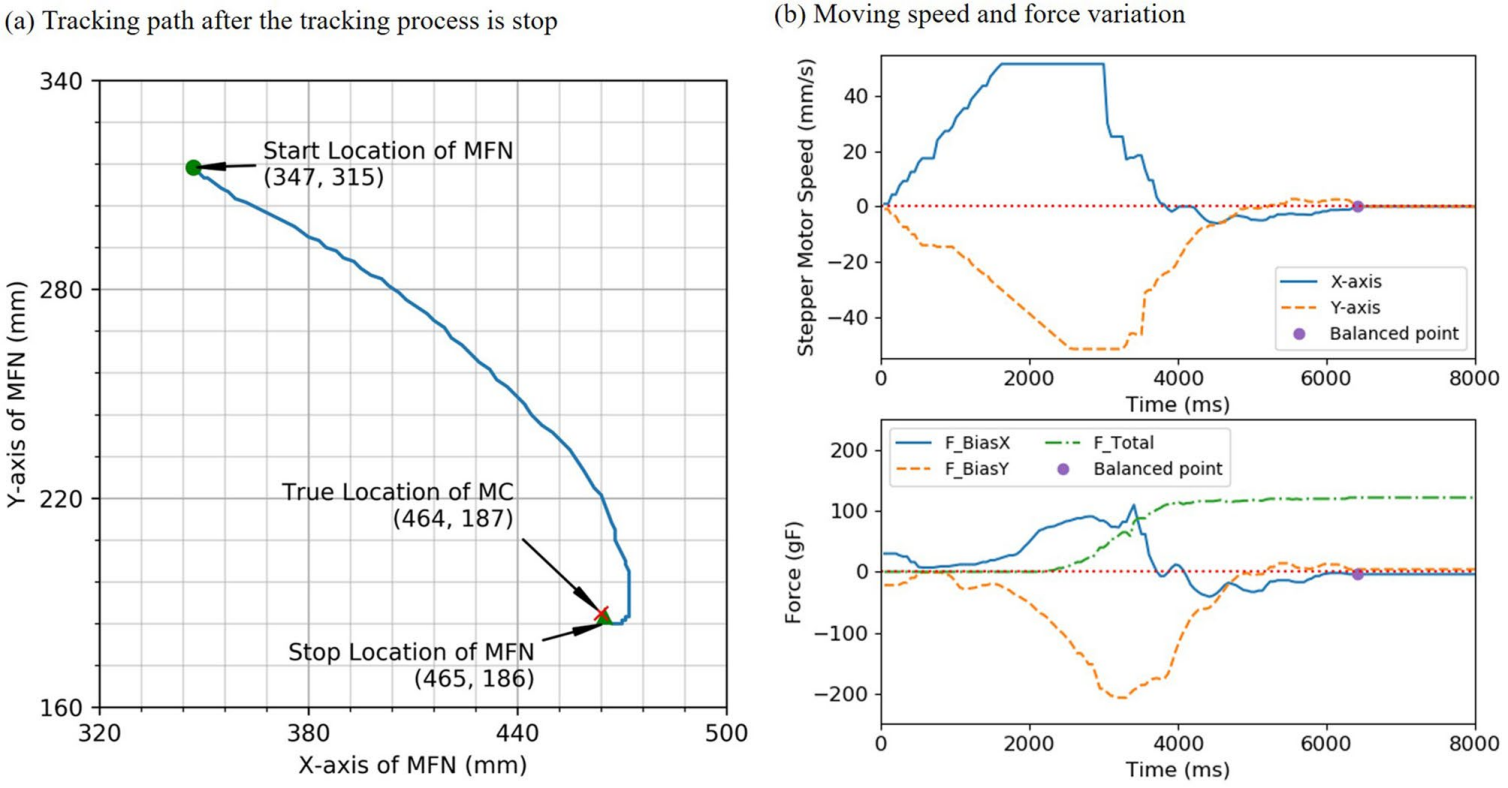
Illustration of the trace, moving speed and force variation in localization function. (**a**) The tracking path illustrates a localization trace after the tracking process is stopped. The starting point of EPM was placed at (x = 347, y = 315), and the IPM was placed at (x = 464, y = 187) with a vertical height difference of 7.5 cm. (**b**) The variation in motor speed and force vectors are presented.

Figure 3a illustrates a localization trace after the tracking process is stopped, where the system does not know the location of the IPM at the beginning. As shown in Fig. 3b, the variation in motor speed and force vectors are presented. The maximum speed of the stepping motor was 51.54 mm/s during localization and the force bias coverage to the balanced state in an instant. Overall, the tracking process required 6.49 s for the EPM to reach the destination (x = 465, y = 186) resulting in 175 mm of displacement with a localization error of 1.41 mm. Table 2 lists the positioning error with six vertical height differences between EPM and IPM. The results show the accurate locating and are as well or better than the errors reported in the articles^15–17,26^ in the xy-domain (see Table 3).

**Table 2:** Average tracking accuracy in different vertical distances from EPM to MC.

Vertical distance from EPM to MC (cm)	Δx (mm)	Δy (mm)
5	0.59 ± 0.51	0.53 ± 0.39
6	0.76 ± 0.32	0.56 ± 0.32
7	0.97 ± 0.50	1.12 ± 0.47
8	0.99 ± 0.42	1.72 ± 0.34
9	1.47 ± 0.39	2.74 ± 0.32
10	2.07 ± 1.45	3.18 ± 0.88

The average accuracy presented in mean error ± standard deviation.

**Table 3:** Average accuracy of previous methods.

Methods	Δx (mm)	Δy (mm)
Salerno et al.^26^	− 3.2 ± 18	5.4 ± 15
Di Natali et al.^15^	− 3.40 ± 3.2	− 3.80 ± 6.2
Taddese et al.^17^	1.56 ± 1.41	4.1 ± 1.67

	Δr (mm)	Δ*θ* (°)
Di Natali et al.^16^	6.2 ± 4.4	1.12 ± 0.47

The average accuracy presented in mean error ± standard deviation.

### Autonomous navigation: simulation in synthetic colon

**Figure 4:**
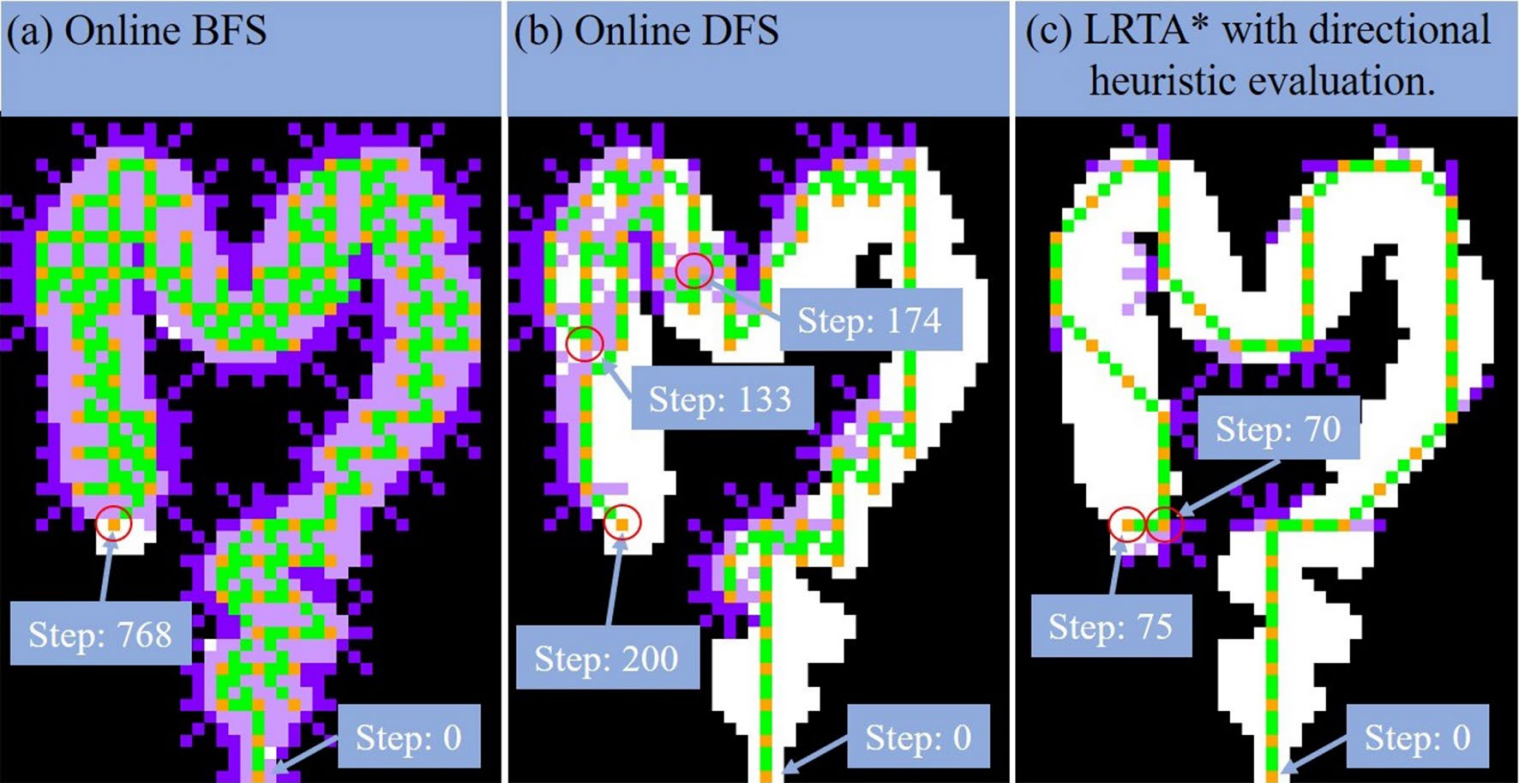
The trajectory simulation in synthetic colon with (**a**) online BFS (**b**) online DFS (**c**) LRTA* method. The taken steps for online BFS, online DFS and LRTA* are 768, 200 and 75, respectively. The non-passable states and the passable states are represented in black and white, respectively. When the algorithm starts to explore, the state in each iteration and the trajectory between states are represented in orange and green, respectively. If the non-passable state is visited, the trajectory is shown in purple.

The simulation trajectories of three path planning algorithms using a synthetic colon map are shown in Fig. 4. The taken steps for LRTA*, online depth-first search (DFS) and online breadth-first search (BFS) are 75, 200 and 768, respectively. The LRTA*’s path with our directional heuristic evaluation design shows a tremendous improvement to other methods not only in route complexity but less intermediate points. In the DFS simulation, it’s worth noting that the inflexible traveling sequence may step backward like what happened at the 133-th step and turning back at the 174-th step. As a result, we choose LRTA* for our synthetic colon autonomous navigation.

### Autonomous navigation: experiments in a highly realistic colonoscopy training model

**Figure 5:**
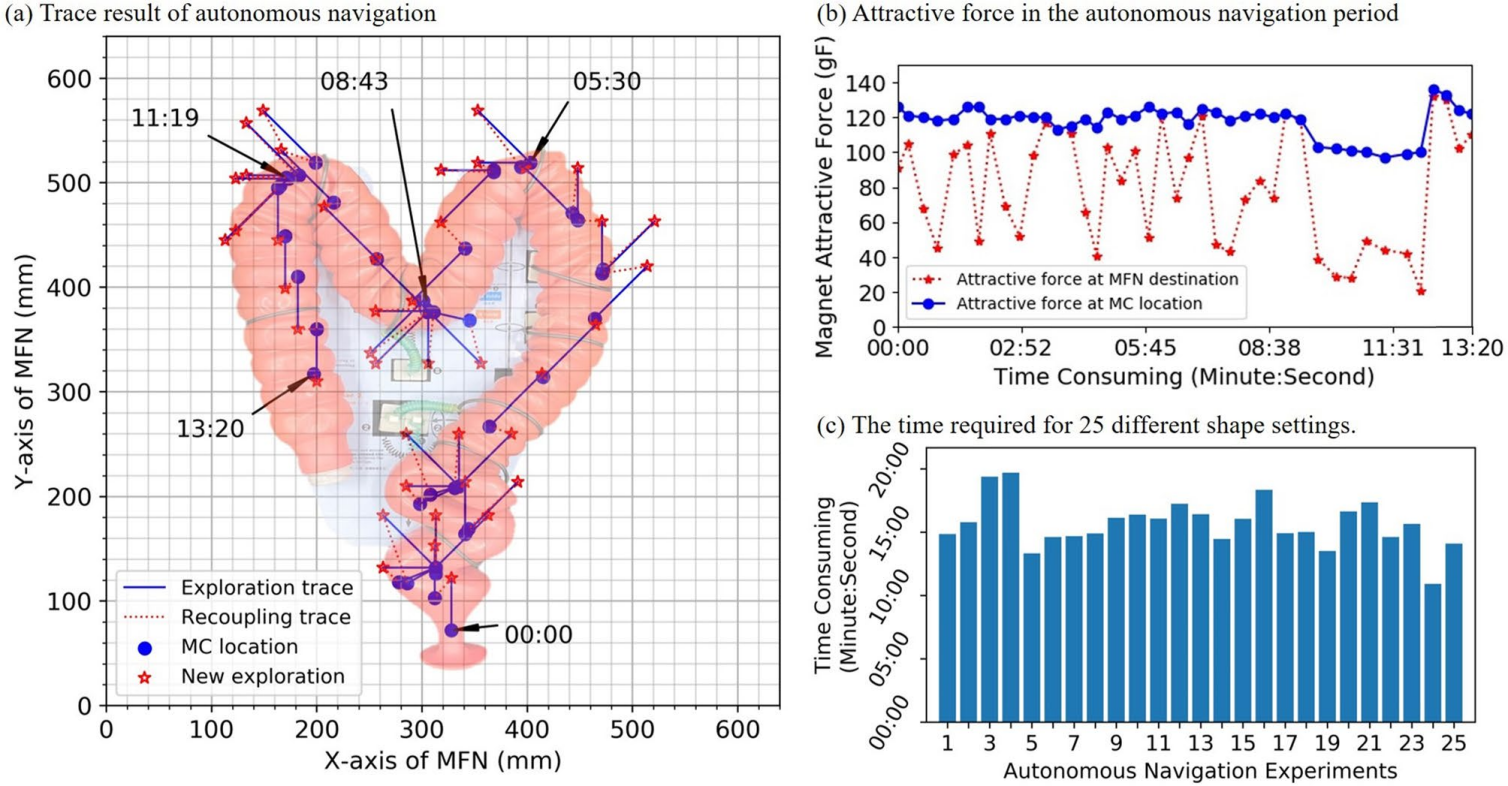
The tracking result of autonomous navigation. (**a**) Illustration of the autonomous navigation trajectory. (**b**) Attractive force in each state corresponds to the tracking result in the graph (**a**). (**c**) Time required to complete autonomous navigation experiment 25 times. The average navigation duration was 15 min 38 s, corresponding to an average moving speed of 96 mm/min.

As shown in Fig. 5a, the EPM guides the IPM in steps of 5 cm along the trajectory generated by LRTA*. The entire experiment on autonomous navigation was completed in 13 min 20 s, with an average speed 90 mm/min (see Supplementary Video 1). Figure 5b presents the attractive forces generated between the EPM and IPM during autonomous navigation. The red stars in Fig. 5b indicate the attractive force measured after the EPM had moved 5 cm along its current trajectory, whereas the blue dots indicate the attractive force when the EPM was located above the IPM. Obviously, the attractive force is proportional to the distance between the EPM and IPM. Thus, any sustained decrease in attractive force is an indication that the movement of the IPM toward the EPM has been hindered by an obstacle, such as intestinal folds or walls. An example of interference from intestinal folds can be seen in Fig. 5b, as indicated by the large discrepancy between the blue points and red stars during the interval 9:11–12:31.

The intubation rate of the autonomous navigation presented in this paper is 83.33% by 30 experiments and only 5 of them take more than 20 min. As shown in Fig. 5c, the average time required to complete navigation of a training model with 25 shape settings was 15 min 38 s, which represents an average moving speed of 96 mm/min. All 25 experiments are completed without intervention by the operator. Ultimately, these experimental results demonstrate the efficiency of the proposed MAC system.

## Discussion

Based on the advantages and future prospects of autonomous navigation in colonoscopy, researchers in this field have been active a lot in recent years. Trovato et al. developed a screw-type colon endoscope robot with automated motion adjustment controlled via reinforcement learning in vitro and in vivo; however, forward motion through straight segments was slow (11 mm/min) and navigation through bends proved awkward^10^. Ji-Yoon Kim et al. developed a twistable threaded mechanism capable of spiral motion at roughly 350 mm/min^13^. A control algorithm automatically adjusts the direction of movement and position of the robotic arm to maintain the capsule within the arc-shaped tract model. Nonetheless, their model is somewhat unrealistic due to a lack of wrinkles and other impediments encountered in real-world situations. Alsunaydih et al. developed a capacitive-based pressure monitoring system to enable the autonomous navigation of capsule endoscopes^11^. Unfortunately, the PVC tube used to mimic the GI tract in that study did not accurately represent real-world conditions. Prendergast et al. developed an autonomous robotic endoscope’ however, their image-based path search scheme was hindered by indiscrete edges and the irregular shapes of haustral folds^14^. Martin et al. developed a semi-autonomous navigation strategy; however, the user needs to override the system’s choice optionally to select a correct output of orientation^12^. In other words, lumen detection could be unstable due to the sharp turns especially in the rectum. Also, images could be overexposed and even no lumen in sight that needs intervention. Therefore, we developed a non-image-based navigation scheme with a high intubation rate in a fully automated way.

In the current field, many researchers developed the MAC system that used an EPM to guide permanent magnets embedded within a capsule endoscope^27–30^. The magnetic navigation in MAC system provides more friendly procedure instead of the rear-push mechanical actuation in the conventional colonoscopy^30^. In doing so, the patient pain caused by the looping of the endoscope^31^ can be avoided. Note however that variability in the magnetic forces generated by a large-gradient EPM has been shown to hinder the accurate control of slaved devices and make it difficult to predict the forces exerted on tissue^21^. Furthermore, the position of IPM is estimated using a common localization method based on magnetic field strength from a Hall sensor array, which is susceptible to interference from other magnetic fields^32^. Shi et al. sought to overcome the problem of locating the slave magnet under conditions of external magnetic interference by treating the EPM signal as a priori knowledge^33^. In recent works, we sought to resolve this problem through the use of iterative algorithms tasked with identifying and locating the EMP and slave magnet^34^. Nonetheless, localization is still prone to uncertainty in instances where the magnetic moment ratio between magnets is large. The derived magnetic moments^34^ of the proposed EPM and IPM are 388 A m^2^ and 1.857 A m^2^, respectively. The enormous difference in magnetic moments between two magnets resulted in a ratio of 209:1 causes a scaling problem in numerical computation. In this scenario, the conventional approach to multiple magnet localization would not provide positioning results of high accuracy due to the overwhelming influence of the EPM rendering variations in the signal from the slave magnet imperceptible^34^. As a result, most existing methods based on Hall array structure have been implemented only in situations where the magnets possess a similar magnetic moment in the current study. Salerno et al. presented a positioning method based on a triaxial magnetic sensor inside the capsule in the MAC system^26^. The method overcomes the limitation of the location where the magnetic moment ratio between magnets is large. Di Natali et al. improved the system with several Hall sensors and an inertial measurement sensor installed in the capsule to enable real-time positioning method^15^, and the efficiency improvement was also provided^16^. Taddese et al. present a hybrid approach^17^ that solves the singularity problem based on the articles^15,16,26^. However, these methods complicate structure of the endoscope and the complex algorithm calculation is needed. In addition, due to frequent contact with tissues in the GI tract during endoscopy, there is a risk of subsequent infection from patient-to-patient transmission^20^. The data show that highly resistant bacteria still exist in 30% or more of reprocessed endoscopes that have undergone high-level disinfection^35^. The report^18^ stated that the infection rate of 115,243 patients 30 days after sigmoidoscopy and colonoscopy was 0.37%, which was higher than the 0.04% of the control group. The article^19^ also shows that there are 1.1% and 1.6% of the postendoscopic infection per 1000 procedures after screening colonoscopy and non-screening colonoscopy, respectively. Postendoscopic infections are more frequent than commonly expected. Given these points, these disadvantages have prompted us to develop the force-based sensing system and use the endoscope designed for disposable.

In the current research, several experiments demonstrated the feasibility and effectiveness of the proposed autonomous navigation system. The detectable range, maximum attraction monitoring and positioning accuracy show the feasibility of tracking function. The attractive force of magnets is inversely proportional to the square of the distance between two magnets; therefore, it was important to consider the measurable range of load cells tracking function. Note that the operating range corresponds to the degree of attraction between the EPM and IPM. If the two devices were too far apart, then the tracking function would neither operate sensitively nor possibly converge to the correct location. If the two devices were too close together, then the resulting attractive forces might be sufficient to cause damage to intestinal tissue between them. Therefore, we outline two experiments aimed at evaluating the feasibility of the proposed system in terms of the maximum attractive forces and a detectable range of the positioning system. Although there are insufficient sensors in the disposable MC and no more information about the location is acquired, the tracking results are still sufficient to fulfill autonomous navigation in subsequent experiments. In future work, the three-dimensional positioning system will be implemented in force sensors and the disposable MC will maximize the value of disposable colonoscopy. Results demonstrate that the positioning accuracy and the detectable range are within an acceptable range. Experiments of autonomous navigation also show the effectiveness of the proposed methods by integrating force-based sensing technology and directional heuristic evaluation in LRTA* algorithm. Moreover, the real-time monitoring of attractive force can be used as a proxy by which to estimate discomfort and the likelihood of damaging intestinal tissue.

## Conclusion

This paper presents a fully autonomous navigation scheme in the cost-effective MAC system via a novel tracking technique using load cells and LRTA* for path searching. The accuracy of load cells tracking function is promising. The average tracking accuracy on the MFN platform in x-axis and y-axis are 1.14 ± 0.59 mm and 1.61 ± 0.45 mm (mean error ± standard deviation). The EPM is able to magnetic recoupling with IPM by minimizing the force bias on x-axis and y-axis. Also, the sensing information can be used for monitoring the attractive force of the IPM from being dangerous to the colon wall. Furthermore, LRTA* algorithm with fortified heuristic evaluation considers the directional information to resolve problems associated with the autonomous navigation of magnetic colonoscopes. In autonomous navigation, all 25 experiments are completed without operator intervention. The average time required was 15 min and 38 s, and the intubation rate was 83.33%. Experimental results demonstrated the feasibility and effectiveness of the proposed system.

## Methods

### MAC system

Figure 1 presents the architecture of the proposed MAC system. Basically, the MAC system comprises the following subsystems: magnetic colonoscope (MC), magnetic field navigator (MFN) platform, load cell module.

The MC features a working channel (diameter = 3.2 mm), a high-definition (HD), a complementary metal-oxide semiconductor (CMOS) camera and multiple white LEDs. These components are affixed in N52 magnetized cylindrical ring-shaped NdFeB magnet while the magnet is represented as an interior permanent magnet (IPM). The total length of the MC is 180 cm, including the flexible silicone tube. The MC sends a video stream ($1280 \times 720$ pixels; 30 frames per second (fps)) to the user interface via the receiver. The difference between the proposed MC and conventional colonoscopes is the fact that MC control is implemented using an EPM rather than a hand-controlled unit in which steering force is transmitted physically from the handle to the rotary joint of the colonoscope. This configuration made it possible for users to create a very thin supple MC tube to reduce discomfort during surgery. For the purpose of decreasing postendoscopic infections, the MC is designed as a disposable device.

The MFN platform in our MAC system provides a working space of $650 \times 650 \times 410\;{\text{mm}}^{3}$ (x-, y-, and z-axes) with control covering five degrees of freedom (5-DOF). The end of the MFN robotic arm contains a diametrically magnetized N52 NdFeB permanent magnet (remanence 1.48 T), providing an external magnetic field sufficient to navigate the IPM in the MC (see Table 4). To observe the forces produced by the EPM and IPM, we installed tension/compression load cells providing 50 Newtons (N) of load capacity in a single axial direction (equally on the four sides) between the MFN robotic arm and the EPM (see Fig. 2). The accuracy of the load cell is 2.5 g-force (gF) calibrated by the manufacturer. The load cells return load-related information (in four directions) to the user interface following amplification in accordance with the RS-485 half-duplex protocol.

**Table 4:** Specifications of magnets used in the proposed MAC system.

Permanent magnets	Size	Material	Direction of magnetization	Remanence $B_{r}$
MFN	$\phi 90\;{\text{mm}}\left( {{\text{OD}}} \right) \times \phi 90\;{\text{mm}}\left( {{\text{ID}}} \right) \times 60\;{\text{mm}}\left( {\text{H}} \right)$	NdFeB	Diametrically magnetized	1.437 T
MCC	$\phi 12\;{\text{mm}}\left( {{\text{OD}}} \right) \times \phi 7.6\;{\text{mm}}\left( {{\text{ID}}} \right) \times 24\;{\text{mm}}\left( {\text{H}} \right)$	NdFeB	Diametrically magnetized	1.435 T

### Working principle: force variation from the load cells

In the proposed system, we developed a novel force-based sensing method involving the use of load cells. In addition, tension and compression load cells can respectively be used to measure positive and negative forces. As shown in Fig. 2, we used four load cells (A, B, C, and D) to measure the forces between the EPM and a robotic arm in four directions simultaneously.

**Figure 6:**
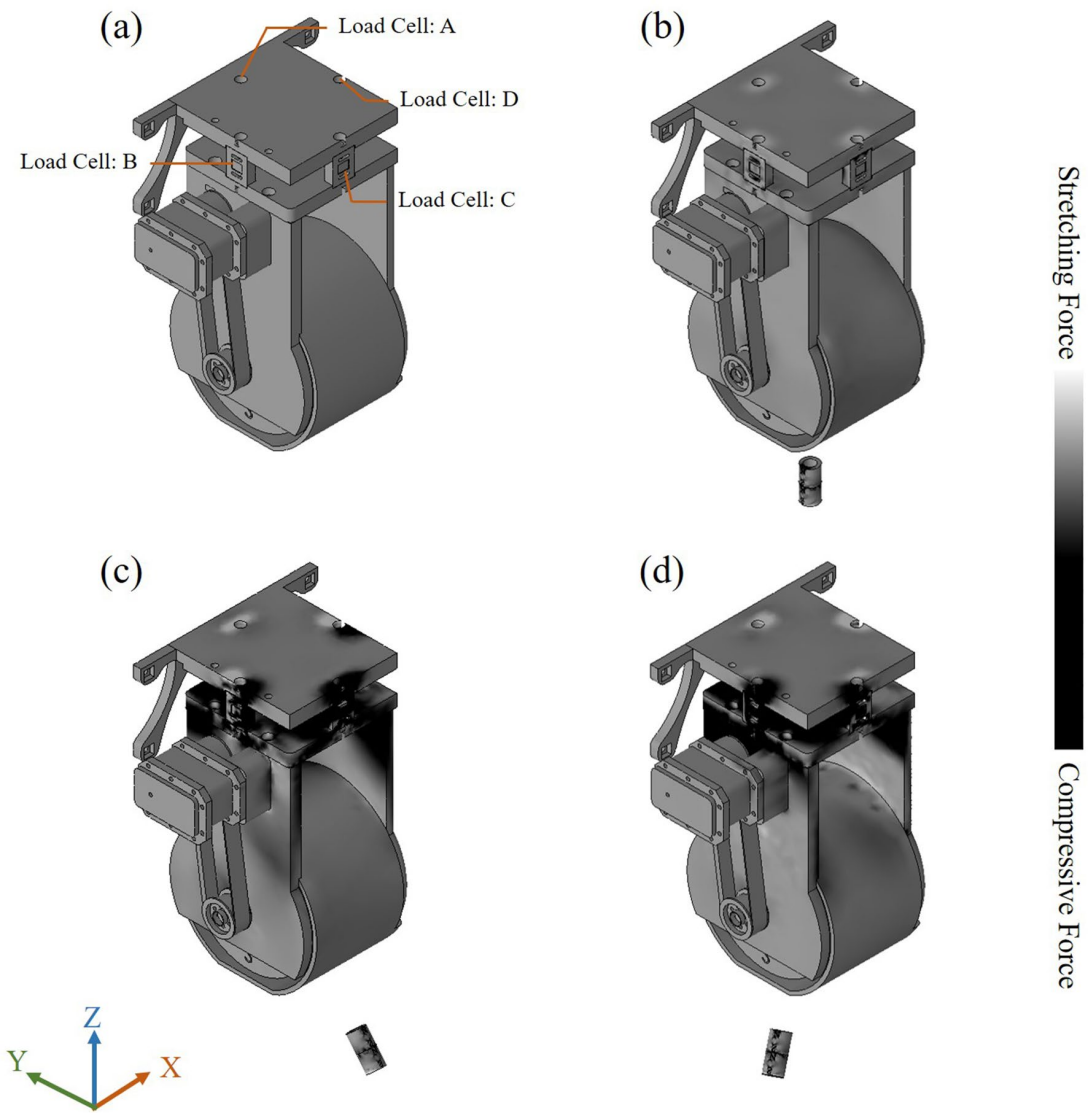
Heat maps showing forces in various locations around an IPM. (**a**) Without magnetic objects; (**b**) IPM directly beneath the EPM; (**c**) IPM is in front of the EPM. Load cell A is stretched while load cell C is squeezed; (**d**) IPM is located in the bottom right region of the EPM. Load cells A and D are stretched while load cells B and C are squeezed.

Magnetic flux from the EPM creates an attractive force to draw the MC. According to Newton’s third law, the EPM is subjected to a force of equal magnitude in the direction opposite to that generated by the IPM. Thus, all of the tensile and compressive forces between the EPM and robotic arm are received by the four load cells. To clarify this description, we drew up various simulation examples using Solidworks (see Fig. 6). When the tracking system is initialized, forces are in a balanced state between the EPM and robotic arm (Fig. 6a), such that the load cells detect nothing. Locating the IPM immediately below the EPM induces the generation of attractive force between the two magnets (Fig. 6b), which produces tension in the load cells but no force bias though. Locating the IPM in front of the EPM produces the stress and strain observed in Fig. 6c. The attraction of the EPM in the forward direction by the IPM causes load cell A to be stretched (positive force) and load cell C to be squeezed (negative force). Finally, locating the magnet in the bottom right of the EPM produces the stress and strain observed in Fig. 6d, wherein load cells A and D are stretched, while load cells B and C are squeezed.

Careful configuration of the load cells makes it possible to use the stretching and compressive forces for IPM positioning. We can obtain the relative directional force vectors between the IPM and EPM in three dimensions using the following equations to calculate:

1 $$\left\{ {\begin{array}{*{20}l} {F_{BiasX} = F_{D} - F_{B} } \hfill \\ {F_{BiasY} = F_{A} - F_{C} } \hfill \\ {F_{{Z{ - }axis}} = F_{A} + F_{B} + F_{C} + F_{D} } \hfill \\ \end{array} } \right.$$

where $F_{BiasX}$, $F_{BiasY}$, and $F_{{Z{ - }axis}}$ are single dimension differential force (bias), indicating a single relative direction from the EPM to the IPM. The force values from load cells A to D are respectively represented as $F_{A}$, $F_{B}$, $F_{C}$ and $F_{D}$.

### Detectable range and maximum attraction of IPM positioning

**Figure 7:**
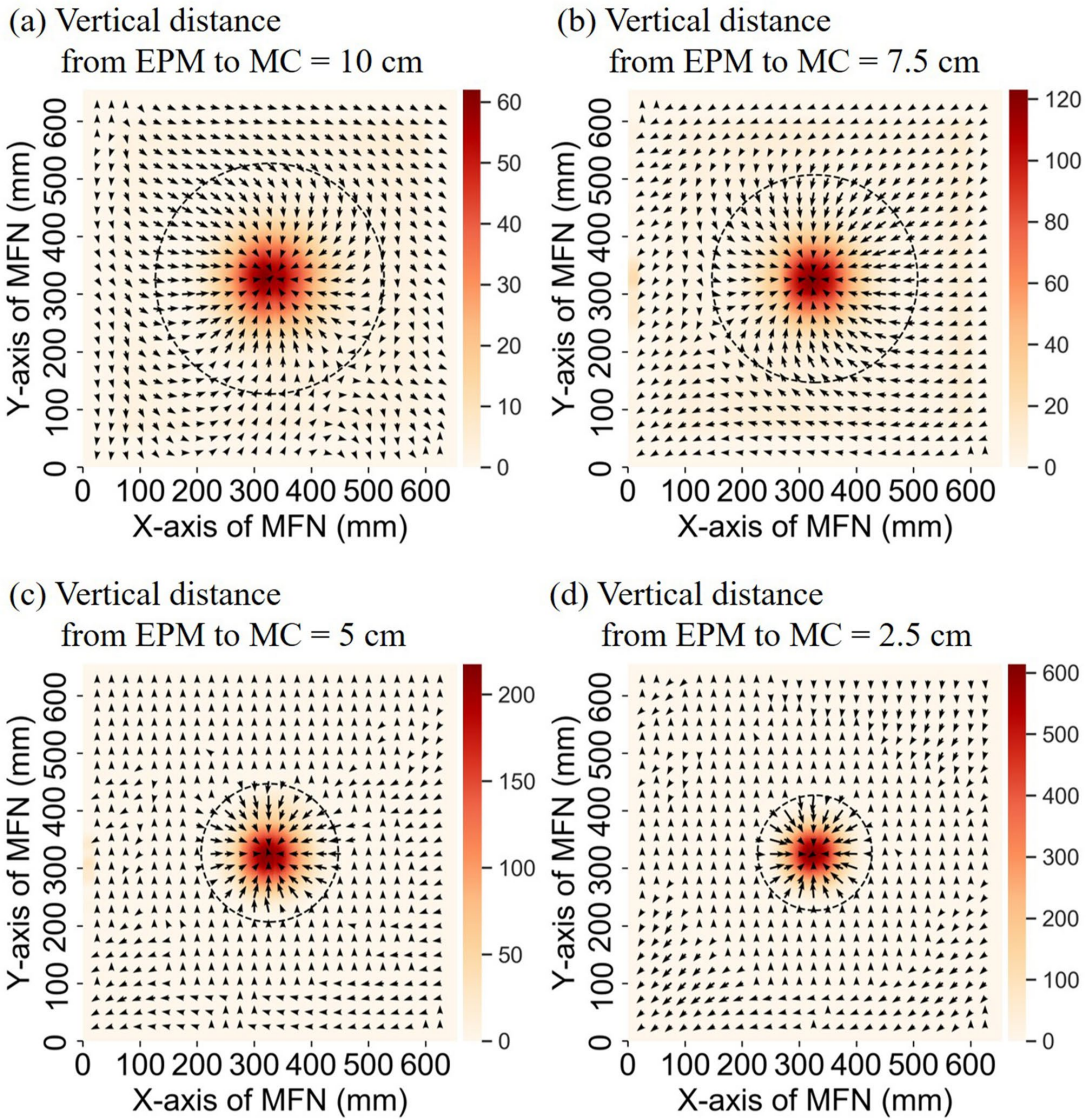
Results of detectable range, force distribution and attractive force when the MC is placed in the center of the MFN platform in four different vertical distance from EPM to MC: (**a**) 10 cm; (**b**) 7.5 cm; (**c**) 5 cm; (**d**) 2.5 cm.

While the IPM was placed at the center of the MFN platform (x = 327, y = 327), the EPM scanned the entire platform continuously at a very slow speed using a stepping motor to avoid unnecessary kinetic forces from interfering with the load cells. Differential force values $F_{BiasX}$, $F_{BiasY}$ and $F_{{Z{ - }axis}}$ were recorded in real-time. Figure 7 presents the detectable range and force distribution with the device suspended at four vertical distances above the model. The arrow indicates the differential force vector in each vertex, as calculated using Eq. (1) in the xy-plane. The dashed circle shows the detectable range in which the robotic arm was able to locate the IPM. The maximum attractive forces and effective detectable range are listed in Table 1.

### Force-based tracking system

From the beginning, $F_{A}$, $F_{B}$, $F_{C}$ and $F_{D}$ will be set to zero when the tracking system is initialized. The load cells tracking function goes into a balanced state when the following conditions $\left| {F_{BiasX} } \right| \le F_{Thres}$ and $\left| {F_{BiasY} } \right| \le F_{Thres}$ are met. The use of load cells in this manner makes it possible to observe the relative direction and magnitude of forces between two magnets in real-time. The actual position of the IPM can be determined using a tracking function involving movement of the robotic arm along the differential vector calculated using Eq. (1) until the difference between $F_{BiasX}$ and $F_{BiasY}$ converges within thresholds and $F_{Z - axis}$ reaches its maximum. The accuracy of the load cell is 2.5 gF. Therefore, we set the threshold force $F_{Thres}$ to 3 gF as stop conditions. As the tracking function is executed, the speeds of the robotic arm moving along the $\omega_{{X{ - }axis}}$ and $\omega_{{Y{ - }axis}}$ are modified instantly in accordance with differential forces $F_{BiasX}$ and $F_{BiasY}$ via the proportional integral (PI) controller as follows:

2 $$\begin{gathered} \omega_{{X{ - }axis}} \left( t \right) = K_{p} F_{BiasX,\;n} \left( t \right) + K_{i} \mathop \sum \limits_{m = 0}^{n} F_{BiasX,\;m} \left( t \right){\Delta }t \hfill \\ \omega_{{Y{ - }axis}} \left( t \right) = K_{p} F_{BiasY,\;n} \left( t \right) + K_{i} \mathop \sum \limits_{m = 0}^{n} F_{BiasY,\;m} \left( t \right){\Delta }t \hfill \\ \end{gathered}$$

where $K_{p}$ is the proportional gain (multiplier term), $K_{i}$ is the integral gain (another multiplier term), and ${\Delta }t$ is the interval between each control iteration; $n$ represents total control iteration. The chosen controller parameters were $K_{p} = 3000$ and $K_{i} = 500$. And they were obtained by using manual tuning from the practical MFN platform so as to perform the best tracking. The speed changes calculated using Eq. (2) allow the robotic arm to move more smoothly and precisely than could be achieved using a constant speed. Eventually, the MFN robotic arm stops at the top of the IPM, verification of which is sent to the operator and a balanced state for load cells is achieved (see Supplementary Video 2). The differential force sensing technique proposed in this paper makes it possible to perform tracking operations under the situation of not knowing the actual IPM position.

We simulated the implementation of the load cells tracking function using the PI controller. The starting point of EPM was placed at (x = 347, y = 315), and the IPM was placed at (x = 464, y = 187) with a vertical height difference of 7.5 cm. During positioning, the MFN robotic arm separately adjusted motor speeds along the x-axis and y-axis using the PI controller from Eq. (2). Figure 3a illustrates a localization trace after the tracking process is stopped and the variation in motor speed and force vectors are presented in Fig. 3b.

### Path planning algorithm

Automating the guidance of the MC requires a MFN path planning algorithm based on artificial intelligence (AI). Note that the algorithm must have the ability to navigate unknown regions in which the path of the intestine varies with each patient in an unpredictable manner^36^ and the target location is unknown at the beginning of the search process. Most search algorithms are based on the known environments that the agent can pre-compute solution before stepping into the environments, also called offline search algorithms. In contrast, the online search algorithm performs the method that interleaves computation and action in an unknown environment. That is, the online search algorithm should take an action first, then observe the environment and finally make the next move. There are two well-known basic traversal methods that can be applied in an unknown environment, breadth-first search (BFS)^37^ and depth-first search (DFS)^38^. However, cumbersome exploration of BFS process makes it inefficient. In contrast, DFS approach is better suited than BFS to navigating the bewildering convolutions of the intestine where there is only one path from the rectum to the cecum. Nonetheless, the initial conditions of DFS stipulate that all directions are tested in the same order of priority in every iteration, which inevitably leads to numerous unnecessary actions.

**Figure 8:**
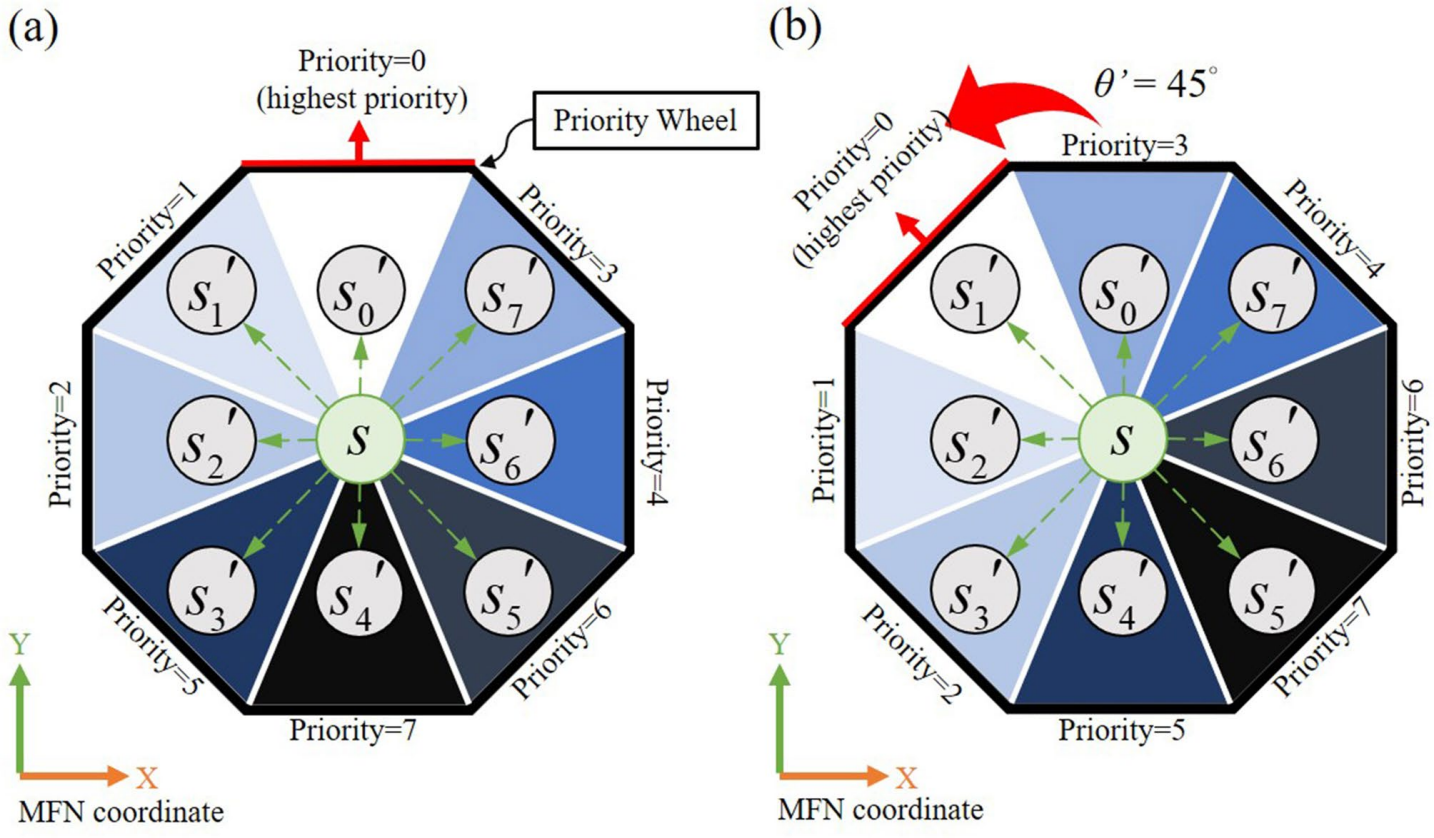
The orchestration of visiting direction sequence in heuristic evaluation design while the passable direction (action) from the previous iteration is (**a**) $\theta^{\prime } = 0^{^\circ }$ and (**b**) $\theta^{\prime } = 45^{^\circ }$.

In the current study, we apply the LRTA* method^39^ with designed directional heuristic evaluation to overcome the inefficiency of DFS that is more flexible to the synthetic colon. In the design of heuristic evaluation, the passable direction (action) from the previous iteration is adopted to change the current state neighbors’ heuristic costs with a specific priority. Considering common intestinal shape and trends, the neighbors’ heuristic value from the current state are designed proportionally with direction priority sequence defined in Fig. 8a (initially $\theta^{\prime } = 0^{^\circ }$). The $s$ is the current state and $s_{i}^{\prime }$ refers to the next state of *i*-th direction; $\theta^{\prime }$ indicates the previous yaw angle of the EPM. While the previous yaw angle changed to $\theta^{\prime } = 45^{^\circ }$ for instance, the evaluation will rearrange the priority by rotating the priority wheel for $\theta^{\prime }$ degrees to make the searching process more flexible, as shown in Fig. 8b. A simple embellishment of heuristic evaluation allows the LRTA* algorithm to optimize the next state for the following step based on local trends in the yaw angle $\theta^{\prime }$ of the previous (reference) path and thereby avoid the need for unnecessary searches.

In order to verify the algorithm, three path planning algorithms were simulated with high-level dynamic programming language Python with version 3.6.8. To generate the digital architecture of the synthetic colon, the synthesized colon image is generated by taking photos of the realistic colonoscopy training model placed on the MFN platform and 10 times scaling down. After that, the features of the intestinal tract model are expressed as a passable state, and other areas are expressed as impassable states, which are represented in white and black, respectively. By applying the BFS, DFS and LRTA* with designed directional heuristic evaluation, the taken steps of simulation results are 75, 200 and 768, respectively. As shown in Fig. 5, we choose LRTA* for our synthetic colon autonomous navigation.

### Autonomous navigation control strategies

**Figure 9:**
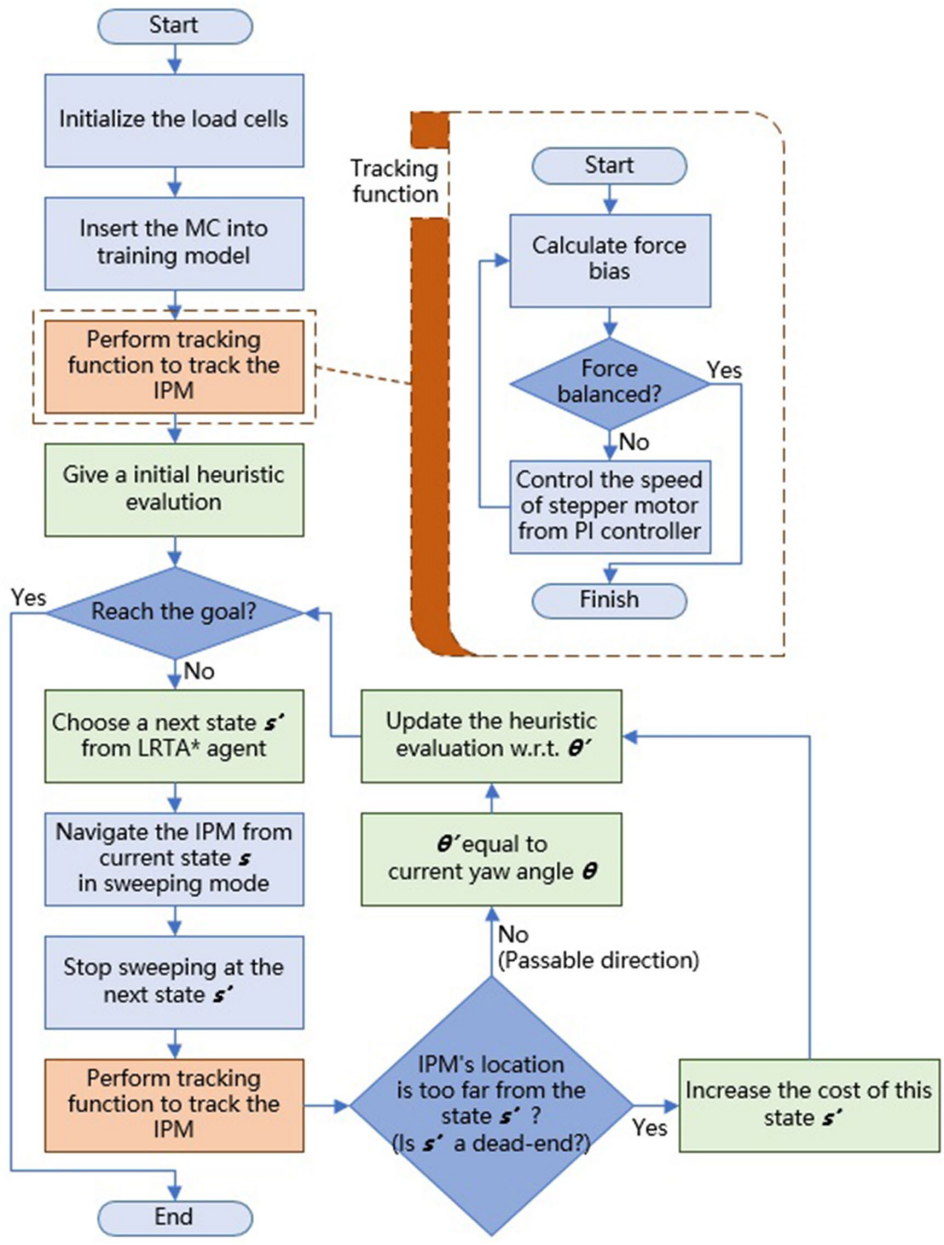
The flowchart of the autonomous navigation in the MAC system.

The integration of autonomous navigation control strategies with this search scheme and force-based sensing makes it possible to implement autonomous navigation in the MAC system. Figure 9 illustrates the implementation of LRTA* scheme within the MAC system for autonomous navigation, where the $\theta$ is the yaw angle towards the next state $s^{\prime }$. While the exploration process is started, IPM is dragged more than one-half of the total distance moved by the EPM, then the algorithm treats the direction (action) and target state in this iteration as passable. In contrast, if the IPM is dragged less than one-half of the total distance moved by the EPM, then the algorithm treats the direction (action) and target state in this iteration as a dead-end. The algorithm then initiates an exploration process until the function of LRTA* finishes or is interrupted by the user. The search algorithm does not have any information about the graph or destination location; therefore, it is up to the operator to determine whether the end location has been reached (based on the MC video) and manually halt the process.

The 30 experiments of autonomous navigation were implemented to demonstrate the efficiency and feasibility of the proposed MAC system. In these experiments, 5 cm was the shortest distance that the EPM had to move in a single step. When using steps of less than 5 cm, the combined friction and flexibility of the intestine walls prevented the IPM from being dragged by the EPM, such that it was often left behind. Once the EPM tracked down the IPM based on the differential force vector, it positioned itself directly above (see Supplementary Video 1).

Considering the effectiveness of practical application, the intubation rate is defined according to the time spent in the experiment. According to the published definition of cecal intubation time benchmark^40^, the minimal competency range is 15 to 20 min. Therefore, the definition of successful intubation is the cecal intubation time less than 20 min. In this research, 30 experiments were conducted and only 5 of them take more than 20 min to reach. The average time in 25 autonomous navigation experiments is 15 min 38 s while the intubation rate is 83.33%. One of the experiments shows the navigation trajectory of MC and attractive forces after reaching the cecum, as shown in Fig. 6a,b.

### Experimental setup

**Figure 10:**
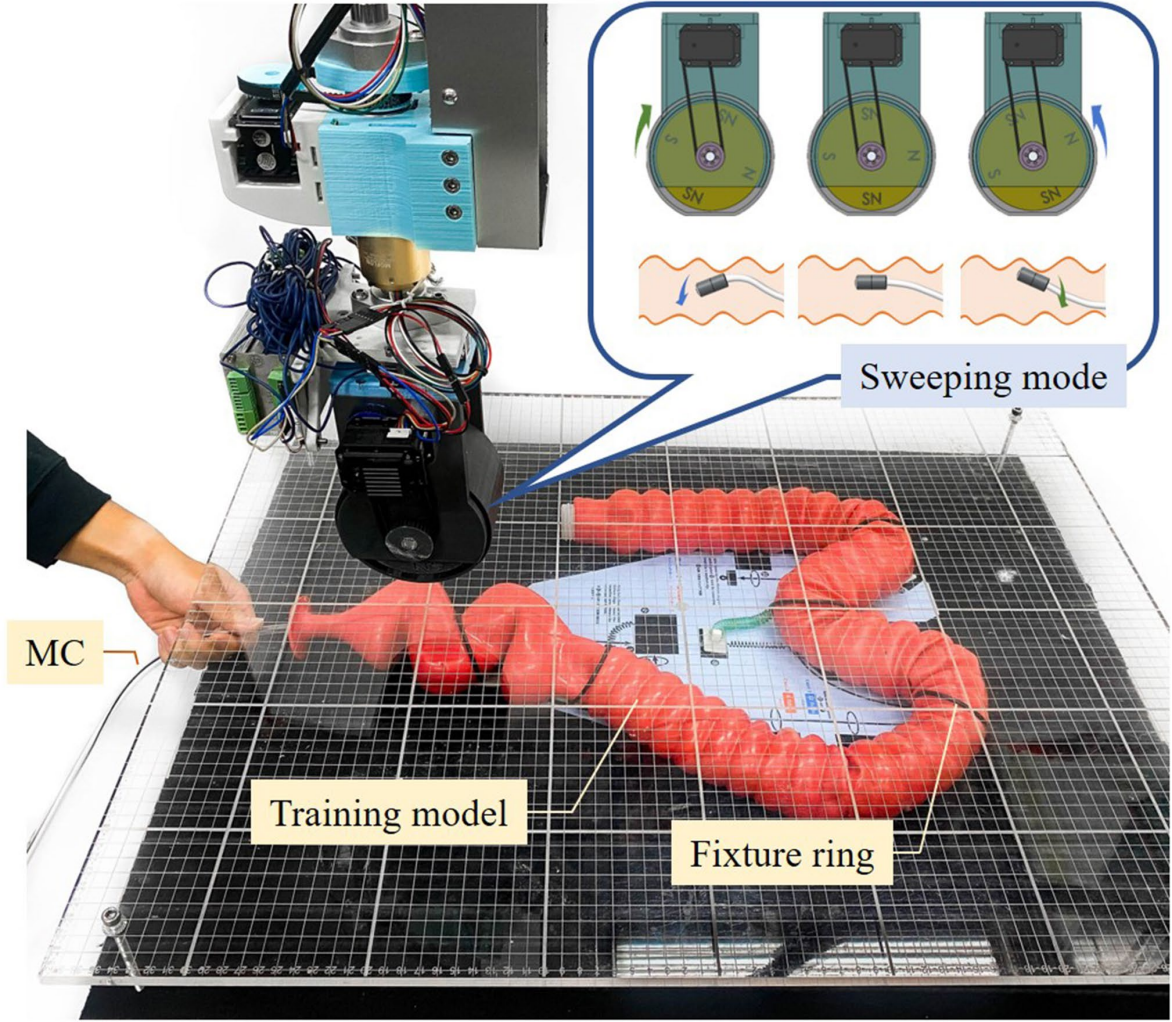
Experimental setup. A flexible colonoscope training model was placed on the MFN platform and covered with a clear acrylic board. While the EPM is guiding the MC, the illustration of EPM in sweeping mode is also presented.

Figure 10 presents the experimental setup used in this study. We simulated the environment of a human intestine using a 150 cm colonoscope training model (KKM40; KYOTO KAGAKU CO). By fixing individual sections, the colonoscope training model can be arranged in a variety of shapes simulating inter-patient differences. Prior to the experiment, the colonoscope training model was placed on a table and covered with a transparent acrylic board. To ensure smooth operations, lubricant was initially applied to the MC. The MC was manually inserted for a short distance, after which the operator only supported the weight of the tube (i.e., did not apply propulsion). We implemented EPM in sweeping mode to produce a sweeping magnetic field, wherein the pitch angle of the magnet is rotated between − 45° and 45°. This was done to ensure smooth passage past intestinal wrinkles.

## Supplementary Information

Supplementary Video 1.

Supplementary Video 2.

Supplementary Information 1.

## Abbreviations

AI: Artificial intelligent
CRC: Colorectal cancer
GI: Gastrointestinal
CE: Capsule endoscopy
MAC: Magnetic-assisted colonoscopy
EPM: External permanent magnet
IPM: Interior permanent magnet
DFS: Depth-first search
BFS: Breadth-first search
MFN: Magnetic field navigator
MC: Magnetic colonoscope
HD: High-definition
CMOS: Complementary metal-oxide semiconductor
LRTA*: Learning real-time A*
fps: Frames per second
PI: Proportional integral
N: Newtons
